# OSA and Acute Coronary Syndrome Severity: The Potential Role of Coronary Collateral Circulation

**DOI:** 10.1016/j.chest.2026.02.015

**Published:** 2026-03-07

**Authors:** Krish Dodani, Albina Aldomà, Adriano Targa, Manuel Sánchez-de-la-Torre, Alicia Sánchez-de-la-Torre, Lucía Pinilla, Jorge Abad, Olga Mínguez, Lydia Pascual, Dolores Martínez, Rafaela Vaca, Ivan Juez-Garcia, Ferran Barbé, Ivan D. Benitez

**Affiliations:** a Department of Basic Medical Sciences, University of Lleida, Lleida, Spain; b Translational Research in Respiratory Medicine Group, Hospital Universitari Arnau de Vilanova-Santa Maria, Institut de Recerca Biomèdica Lleida, Lleida, Spain; c Cardiology Department, Hospital Tortosa Verge de la Cinta, Institut de Recerca Biomèdica Catalunya Sud (IRBCatSud), Tortosa, Spain; d El Centro de Investigación Biomédica of Respiratory Diseases, Institute of Health Carlos III, Madrid, Spain; e Precision Medicine in Chronic Diseases Group, Hospital Nacional de Parapléjicos, Instituto de Investigación Sanitaria de Castilla-La Mancha, Toledo, Spain; f Department of Nursing, Physiotherapy and Occupational Therapy, Faculty of Physiotherapy and Nursing, University of Castilla-La Mancha, Toledo, Spain; g Respiratory Department, Hospital Universitari Germans Trias i Pujol, Badalona, Barcelona, Spain; h Sleep Health and Adelaide Institute for Sleep Health, Flinders Health and Medical Research Institute, College of Medicine and Public Health, Flinders University, Adelaide, SA, Australia

**Keywords:** acute coronary syndrome, angiogenesis, cardiovascular disease, coronary collateral circulation, myocardial damage, OSA

## Abstract

**Background:**

Some evidence suggests that OSA may attenuate myocardial injury and reduce infarct severity during acute coronary syndromes (ACSs), potentially through stimulation of coronary collateral circulation (CCC). However, the relationship between OSA and collateral development, and their combined impact on ACS event severity, remains unclear.

**Research Question:**

What is the relationship between OSA and coronary collateral development and what is their combined impact on ACS severity?

**Study Design and Methods:**

Post hoc analysis of the Impact of Sleep Apnea in the Evolution of Acute Coronary Syndrome: Effect on Intervention With CPAP (ISAACC) trial, including patients with first-time ACS with CCC assessment using the Cohen-Rentrop scale (CRS), where a score of ≥ 2 (CRS grade 2 or 3) indicated well-developed collaterals. OSA was diagnosed via respiratory polygraphy performed within 24 to 72 hours of hospitalization. Associations between OSA and CRS grade 2 or 3 were evaluated using adjusted logistic regression. Peak creatine kinase (CK), peak cardiac troponin I (cTnI), and left ventricular ejection fraction (LVEF) were analyzed as ACS severity markers.

**Results:**

Of 185 participants included, 79.5% had OSA. Participants were mainly middle-aged male individuals and demonstrated a high comorbidity burden. OSA was associated with greater odds of well-developed collaterals with an adjusted OR of 2.84 (95% CI, 1.24-7.19; *P* = .019), with a dose-response increase across OSA severity categories. Among patients with OSA, the presence of robust collaterals was associated with significantly lower peak CK and cTnI levels during the ACS episode, but not with improved LVEF.

**Interpretation:**

Our research shows that in patients with first-time ACS, OSA was associated with a higher prevalence of well-developed coronary collaterals. Among patients with OSA, robust collateralization was associated with less myocardial injury during ACS, supporting the hypothesis that OSA may promote adaptive vascular remodeling, which warrants further mechanistic investigation.

**Clinical Trial Registration:**

ClinicalTrials.gov; No.: NCT01335087; URL: www.clinicaltrials.gov

FOR EDITORIAL COMMENT, SEE PAGE 303

**Take-Home Points**

**Research Question:** Is OSA associated with increased prevalence of enhanced coronary collateral circulation among patients with acute coronary syndrome (ACS) and what is the impact of coronary collaterals in ACS severity among patients with OSA?

**Results:** Among patients with ACS, increased prevalence of well-developed coronary collaterals was associated independently with OSA severity.

**Interpretation:** The increased prevalence of well-developed collaterals among patients with ACS and OSA and the reduced myocardial damage found among patients with OSA with extensive collateralization imply a potential cardioprotective role of OSA in short-term ACS prognosis.

OSA is characterized by repeated episodes of total (apneas) or partial (hypopneas) upper airway obstruction during sleep, leading to recurrent cycles of intermittent hypoxia, sleep fragmentation, sympathetic overactivity, and oxidative stress. These pathophysiologic consequences have been linked to increased cardiovascular morbidity and mortality.^1^^,^^2^ However, the relationship between OSA and cardiovascular health is complex.^3^ Although robust evidence identifies OSA as a risk factor for cardiovascular disease,^4^ some studies paradoxically report more favorable outcomes in specific cardiovascular contexts.^5^^,^^6^

Prior studies have reported results suggestive of reduced acute coronary syndrome (ACS) severity among patients with OSA. Authors of these studies suggest that the cardioprotective effect of OSA may be mediated by the proangiogenic effects of oxidative stress and intermittent hypoxia.^5^^,^^6^ These results suggest that angiogenic stimulation of coronary collateral circulation may represent cardioprotective adaptations conferred by OSA.

Recent evidence suggests that OSA may promote the development of coronary collateral vessels.^7^, ^8^, ^9^ Coronary collaterals are small preexisting vessels capable of expanding in response to myocardial ischemia, serving as natural bypasses that may preserve myocardial perfusion during ACS, potentially attenuating infarct severity.^10^^,^^11^ This mechanism may be particularly relevant given the high prevalence of OSA in populations with ACS, estimated at approximately 50%.^12^^,^^13^

Evidence regarding the relationship between OSA and coronary collateral circulation (CCC) remains inconclusive. Some studies have observed a positive association between OSA severity and the presence of well-developed coronary collaterals. For example, Summerer et al^7^ reported that patients with myocardial infarction and extensively developed collaterals tended to have a higher apnea-hypopnea index (AHI). Conversely, another study reported that among patients with OSA with total coronary occlusions, those with severe OSA were less likely to demonstrate robustly developed collaterals, potentially reflecting distinct physiologic effects across the spectrum of OSA severity.^8^ Furthermore, the impact of well-developed coronary collaterals on ACS severity in patients with OSA remains unclear.

In this study, we investigated the association between OSA and the presence of well-developed coronary collaterals in patients experiencing a first-time ACS. We also examined how collateral formation influences the severity of ACS in patients with OSA, as measured by biochemical and functional markers of myocardial injury.

## Study Design ad Methods

### Study Design

This was a post hoc analysis of the Impact of Sleep Apnea in the Evolution of Acute Coronary Syndrome: Effect on Intervention With CPAP (ISAACC) trial, a multicenter, open-label, parallel, randomized controlled trial (ClinicalTrials.gov Identifier: NCT01335087),^14^ which evaluated the effect of CPAP treatment in preventing secondary cardiovascular outcomes in patients with ACS. Eligible participants were 18 years of age or older, were hospitalized for ACS, and had an Epworth Sleepiness Scale score of ≤ 10. Patients with > 50% central apneas or Cheyne-Stokes respiration were excluded from the study. ACS was defined as an acute presentation of coronary artery disease, encompassing ST-segment elevation myocardial infarction (STEMI) and non-STEMI, as well as unstable angina. The ethics committee of each participating center approved the study (Coordinating Center Identifier: 2010-852), and all participants provided written informed consent. Details on collection of clinical data can be found in e-Appendix 1.

### Diagnostic Sleep Study

All participants underwent a sleep study within 24 to 72 hours of hospital admission, via respiratory polygraphy (Embletta; ResMed) following national guidelines.^15^ Apnea was defined as a ≥ 10-second cessation of airflow, and hypopnea was define as a ≥ 10-second reduction in airflow associated with ≥ 4% oxygen desaturation. The definition of the study groups in the trial was based on the AHI, which represents the average number of apnea plus hypopnea events per hour of sleep recording. OSA was diagnosed as an AHI of ≥ 15 events/h. OSA severity was classified as moderate for an AHI of 15 to 29.9 events/h and severe for an AHI of ≥ 30 events/h. Furthermore, hypoxic burden was calculated as reported by Pinilla et al^16^ in patients with available sleep study records (n = 129).

### Coronary Collateral Assessment

The team of cardiologists participating in this substudy of the trial assessed CCC during patient admission using the Cohen-Rentrop scale (CRS).^17^ In addition, the cardiologist responsible for this article performed a second independent evaluation by retrospectively assessing the available coronary angiograms. A CRS grade 0 represented no visible collateral vessels, CRS grade 1 represented visualization of vascular channels without filling of the epicardial recipient artery, CRS grade 2 represented partial filling of the epicardial recipient artery, and CRS grade 3 represented complete filling of the epicardial recipient artery. CRS grades 2 and 3 were considered robustly developed collaterals, a classification used to represent collaterals with potential hemodynamic relevance.^18^, ^19^, ^20^ For the present post hoc analysis, only baseline data from patients with first-time ACS with available CCC assessments were analyzed, which were from 2 hospitals, Hospital Universitari Arnau de Vilanova and Hospital Santa María de Lleida.

### ACS Severity Assessment

Systolic function was assessed via left ventricular ejection fraction (LVEF) using Simpson’s biplane method and categorized as preserved (≥ 50%) or reduced (< 50%). Blood samples were collected at admission and every 6 hours from the time of admission until 2 consecutive decreases in cardiac troponin I (cTnI) and creatine kinase (CK) were observed (AccuTnI+3 Beckman-Coulter, UniCel Dxl 600 Beckman-Coulter autoanalyzer, respectively). Peak cTnI and CK were defined as the highest recorded values.

### Statistical Analysis

For descriptive statistics, variables were reported as median (interquartile range) if continuous and as frequency (percentage) if categorical. Differences between groups were assessed via Wilcoxon rank-sum tests for continuous variables and χ^2^ tests for categorical variables.

Associations between OSA severity and presence of CRS grades 2 or 3 were evaluated using unadjusted and adjusted logistic regression models, controlling for age, sex, BMI, as well as hypertension and diabetes for their influence in coronary collateral circulation.^21^^,^^22^ Nonlinear relationships between AHI and collateralization were assessed using generalized additive models. Likewise, the relationship between sleep apnea-specific induced hypoxic burden was assessed via generalized additive models making use of available hypoxic burden records within our study sample.

Among patients with OSA, the association between CRS status and markers of ACS severity was analyzed using single and multivariable linear models for peak cTnI, CK, and LVEF and logistic regression for reduced LVEF (LVEF < 50%). Adjustment of ACS severity models included the same variables as those used for OSA associative models.

All analyses were conducted using R version 4.3.1 software (R Foundation for Statistical Computing). The package mgcv^23^ was used for generalized additive modelling of CRS status as a function of AHI.

## Results

### Baseline Characteristics

Of the 478 patients who were enrolled in the ISAACC study for a first-time ACS, 185 patients had available CCC assessments and were included in the present analysis (e-Fig 1). Overall, no statistically significant differences were observed between included and nonincluded patients in clinical parameters, except for slight differences in OSA severity and in antihypertensive medication use (e-Table 1). The study population consisted primarily of middle-aged male individuals who were overweight with a high burden of comorbidities, consistent with the overall ISAACC population. A total of 147 patients (79.5%) had a diagnosis of OSA with median AHI of 26.8 (interquartile range, 19.4-41.7), of whom 57.8% had moderate OSA and 42.2% had severe OSA. Participants with and without OSA showed similar sociodemographic and anthropometric characteristics (Table 1). Comorbidity profiles and the proportion of patients receiving antihypertensive medications were similar between groups.

**Table 1:** Baseline Characteristics According to OSA Group

Variable	All Patients (N = 185)	Patients Without OSA (n = 38)	Patients With OSA (n = 147)	*P* Value
Sociodemographic characteristics				
Sex				1.000
Male	158 (85.4)	32 (84.2)	126 (85.7)	
Female	27 (14.6)	6 (15.8)	21 (14.3)	
Age, y	57.0 (50.0-65.0)	58.0 (50.2-66.0)	57.0 (50.0-64.0)	.654
Smoking history				.988
Never	33 (17.8)	7 (18.4)	26 (17.7)	
Current	114 (61.6)	23 (60.5)	91 (61.9)	
Former	38 (20.5)	8 (21.1)	30 (20.4)	
Anthropometric characteristics				
BMI, kg/m^2^	28.8 (26.5-31.9)	27.2 (25.2-31.0)	29.1 (26.7-32.1)	.065
Neck circumference, cm	40.0 (38.0-43.0)	41.0 (37.5-43.0)	40.0 (38.0-43.0)	.648
Waist circumference, cm	103 (95.0-111)	101 (95.0-108)	103 (96.0-112)	.189
Hip circumference, cm	103 (97.0-109)	102 (94.5-106)	103 (97.0-110)	.206
Comorbidities				
Hypertension	98 (53.0)	20 (52.6)	78 (53.1)	1.000
Diabetes mellitus	50 (27.0)	8 (21.1)	42 (28.6)	.468
Dyslipidemia	107 (57.8)	22 (57.9)	85 (57.8)	1.000
Antihypertensive treatment				
Angiotensin-converting enzyme inhibitor	50 (27.0)	11 (28.9)	39 (26.5)	.925
Angiotensin II receptor blockers	17 (9.19)	3 (7.89)	14 (9.52)	1.000
Statin	46 (24.9)	11 (28.9)	35 (23.8)	.658
Admission parameters				
Admitting diagnosis				.423
STEMI	148 (80.0)	28 (73.7)	120 (81.6)	
NSTEMI	33 (17.8)	9 (23.7)	24 (16.3)	
UA	4 (2.16)	1 (2.63)	3 (2.04)	
Length of stay, d				
Hospital	6.00 (5.00-8.00)	6.00 (4.00-8.00)	6.00 (5.00-8.00)	.361
Cardiology unit	2.00 (2.00-3.00)	2.00 (2.00-2.75)	2.00 (2.00-3.00)	.656
Biochemistry findings				
Glucose, mg/dL	115 (96.0-164)	121 (96.5-148)	114 (95.5-171)	.988
Creatinine, mg/dL	0.88 (0.75-1.05)	0.89 (0.77-1.04)	0.88 (0.74-1.04)	.969
Hemoglobin, g/dL	13.8 (12.9-14.9)	14.0 (13.1-15.3)	13.8 (12.8-14.9)	.306
HbA1c, %	5.70 (5.50-6.43)	5.55 (5.47-5.93)	5.80 (5.50-6.60)	.018
Total cholesterol, mg/dL	176 (155-205)	166 (147-197)	179 (157-206)	.147
Low-density lipoprotein, mg/dL	107 (88.1-134)	103 (83.0-123)	107 (90.0-136)	.463
High-density lipoprotein, mg/dL	41.0 (34.0-46.0)	40.0 (37.0-47.0)	41.0 (34.0-45.8)	.259
Triglycerides, mg/dL	136 (98.0-181)	109 (81.0-143)	141 (102-184)	.012
Platelet, cells/μL × 10^3^	208 (178-245)	192 (155-232)	209 (180-246)	.174
Leukocytes, cells/mm^3^ × 10^3^	10.5 (8.47-12.7)	10.8 (8.87-12.7)	10.3 (8.40-12.6)	.582
Neutrophils, %	76.0 (68.0-83.0)	72.5 (64.5-79.8)	77.0 (68.2-83.0)	.053
Sleep parameters				
Apnea-hypopnea index, events/h	22.0 (16.0-37.0)	7.40 (3.82-11.0)	26.8 (19.4-41.7)	< .001
Oxygen desaturation index, events/h	19.6 (11.2-35.9)	6.20 (2.60-10.3)	24.4 (15.0-39.9)	< .001
T90, %	2.90 (0.30-11.3)	0.30 (0.00-2.70)	3.90 (0.60-16.1)	< .001
Spo_2_, %				
Mean	93.0 (92.0-94.4)	93.4 (92.3-95.0)	93.0 (91.9-94.2)	.098
Minimum	85.0 (80.0-88.0)	88.0 (85.0-90.0)	84.0 (79.0-87.0)	< .001

Data are presented as No. (%) or median (interquartile range). Differences between groups were tested with the Wilcoxon-ranked sum test if variables were continuous and χ^2^ tests if they were categorical. HbA1c = glycated hemoglobin; NSTEMI = non-ST-segment elevation myocardial infarction; STEMI = ST-segment elevation myocardial infarction; T90 = percentage of total sleep recorded time spent at < 90% oxygen saturation; UA = unstable angina.

### Association Between OSA and Coronary Collateral Presence

Robust coronary collaterals (CRS grade 2 or 3) were observed in 41.5% of patients with OSA and in 23.7% of patients without OSA. OSA was associated with higher odds of CRS grade 2 or 3, with an OR of 2.29 (95% CI, 1.04-5.44; *P* = .047). This association strengthened after adjustment for confounding factors such as age, sex, BMI, hypertension, and diabetes, with an OR of 2.84 (95% CI, 1.24-7.19; *P* = .019). A dose-response pattern was observed across OSA severity categories, with an adjusted OR of 2.61 (95% CI, 1.09-6.87; *P* = .039) for moderate OSA and an adjusted OR of 3.22 (95% CI, 1.27-8.90; *P* = .018) for severe OSA (Table 2). When complete rank of OSA severity was analyzed as a continuous variable, a nonlinear association was observed between AHI and likelihood of CRS grade 2 or 3. Specifically, higher likelihood of CRS grade 2 or 3 was observed with increasing AHI, up to approximately 50 events/h. Past this threshold, this increase was lost, although the CIs widened beyond this range because of data sparsity (e-Fig 2). The same relational pattern was observed between hypoxic burden and likelihood of CRS grade 2 or 3 in a subsample with sleep study records available (n = 129), although it did not reach statistical significance (e-Fig 3).

**Table 2:** Associations of OSA Status and OSA Severity With Well-Developed Collaterals

Variable	Unadjusted			Adjusted^a^		
	OR	95% CI	*P* Value	OR	95% CI	*P* Value
OSA status (reference, no OSA)						
OSA (AHI ≥ 15 events/h)	2.29	1.04-5.44	**.047**	2.84	1.24-7.19	**.019**
OSA severity (reference, no OSA)						
Moderate OSA (AHI ≥ 15 events/h and < 30 events/h)	2.15	0.93-5.33	.083	2.61	1.09-6.87	**.039**
Severe OSA (AHI ≥ 30 events/h)	2.49	1.04-6.37	.054	3.22	1.27-8.90	**.018**

Data are ORs for CRS grade 2 or 3 of the level respective of its reference. AHI = apnea-hypopnea index. Bold indicates *P* values ≤ .05.

a Adjusted for age, sex, BMI, hypertension, and diabetes.

### Effect of Coronary Collaterality on ACS Severity Markers in Patients With OSA

**Figure 1:**
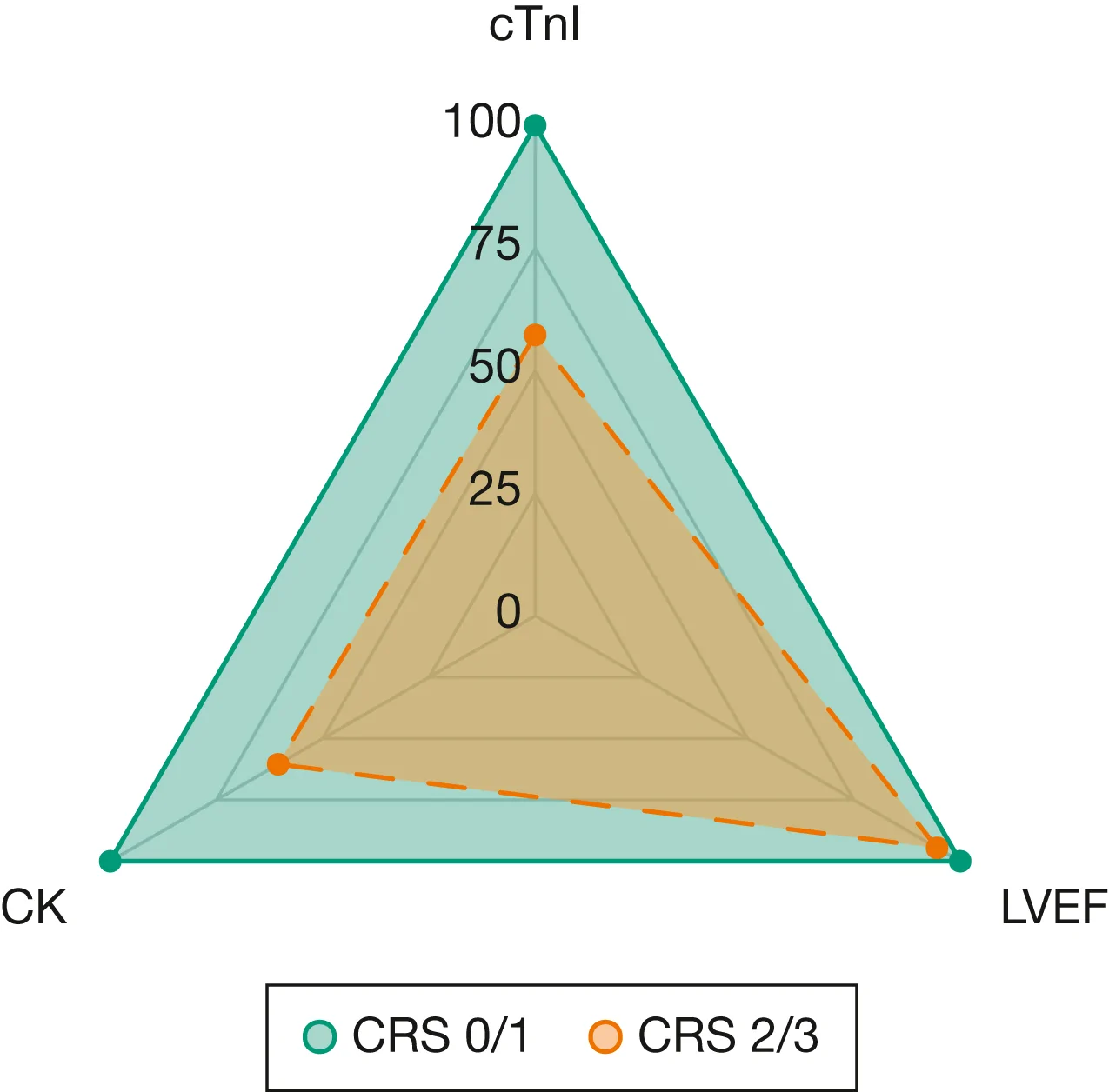
Radar plot depicting the percentage differences in cardiac markers normalized to the CRS grade 0 or 1 group. CRS = Cohen-Rentrop scale; cTnI = cardiac troponin I level; CK = creatine kinase; LVEF = left ventricular ejection fraction.

Among patients with OSA (n = 147), 61 patients (41.5%) had CRS grade 2 or 3. Similar sociodemographic and anthropometric characteristics and comorbidity burden were observed between the CRS grade 2 or 3 and CRS grade 0 or 1 groups (e-Table 2). The results of the association between cardiac biomarkers and collateral circulation are shown in Figure 1 and Table 3. The CRS grade 2 or 3 group showed lower myocardial damage during ACS than the CRS grade 0 or 1 group, with a mean difference in peak cTnI levels of –22.6 ng/mL (95% CI, –38.4 to –6.83 ng/mL). This corresponded to a 42.8% reduction relative to the mean peak cTnI level observed in the CRS grade 0 or 1 group (53 ng/mL). Similarly, the CRS grade 2 or 3 group showed lower values in CK, with a mean difference of –938.03 U/L (95% CI, –1603.11 to –272.93 U/L), which represented a reduction of 39.51% respective of the CRS grade 0 or 1 group (mean, 2,374 U/L). The associations between robust coronary collaterals and myocardial damage were consistent after adjusting for confounders (age, sex, BMI, diabetes, and hypertension). Regarding cardiac function, lower mean LVEF was observed in the CRS grade 2 or 3 group, with a nonsignificant mean difference of –2.99% (95% CI, –6.42% to 0.44%) compared with the CRS grade 0 or 1 group. Furthermore, no statistically significant difference was found in the risk of reduced LVEF (LVEF < 50%) in the CRS grade 2 or 3 group compared with the CRS grade 0 or 1 group, with an adjusted OR of 1.85 (95% CI, 0.928-3.72; *P* = .082; data not shown).

**Table 3:** Infarct Severity Markers Levels in CRS Grade 2 or 3 vs CRS Grade 0 or 1

Variable	Unadjusted			Adjusted^a^		
	Mean Difference	95% CI	*P* Value	Mean Difference	95% CI	*P* Value
Peak cTnI level, ng/mL	–22.6	–38.4 to –6.83	**< .01**	–21.7	–37.9 to –5.5	**< .01**
Peak CK level, ng/mL	–938.02	–1,603.1 to –272.9	**< .01**	–900.9	–1,577.7 to –222.1	**< .01**
LVEF, %	–2.99	–6.42 to 0.44	.087	–3.26	–6.76 to 0.24	.069

CK = creatine kinase; CRS = Cohen-Rentrop scale; cTnI = cardiac troponin I; LVEF = left ventricular ejection fraction. Bold indicates *P* values ≤ .05.

a Adjusted for age, sex, BMI, hypertension, and diabetes.

## Discussion

In this post hoc analysis of patients with first-time ACS from the ISAACC trial, the prevalence of well-developed CCC was associated independently with OSA severity. Among patients with OSA, the presence of robust collaterals was associated with significantly lower biochemical markers of myocardial injury during the ACS episode, but not with improved systolic function.

Collateral recruitment has been shown to be augmented among patients with OSA with total coronary occlusion.^9^ Additionally, a clinical history of OSA was shown to be an independent predictor of robust collaterals among 1,863 patients with STEMI.^24^ The observations of the present study are consistent with these, with the added benefit of counting with an actual OSA diagnosis instead of relying on clinical history. In this study, a quasilinear relationship was observed between AHI and the likelihood of well-developed collaterals, with the likelihood increasing almost to approximately 50 events/h. Same relational pattern between hypoxic burden and well-developed collateral likelihood was observed, suggesting the possible role of intermittent hypoxia severity on well-developed collateral prevalence. Similarly, a study comprising 60 patients with OSA diagnosed via polysomnography experiencing either total coronary occlusion or STEMI reported that patients with severe OSA were less prone to demonstrate well-developed collaterals than those with mild or moderate OSA.^8^ Furthermore, Summerer et al^7^ reported a linear association between OSA severity and coronary collateral development. In their study, they reported an association between total and obstructive AHI and robust collateral presence, but not central AHI.

**Figure 2:**
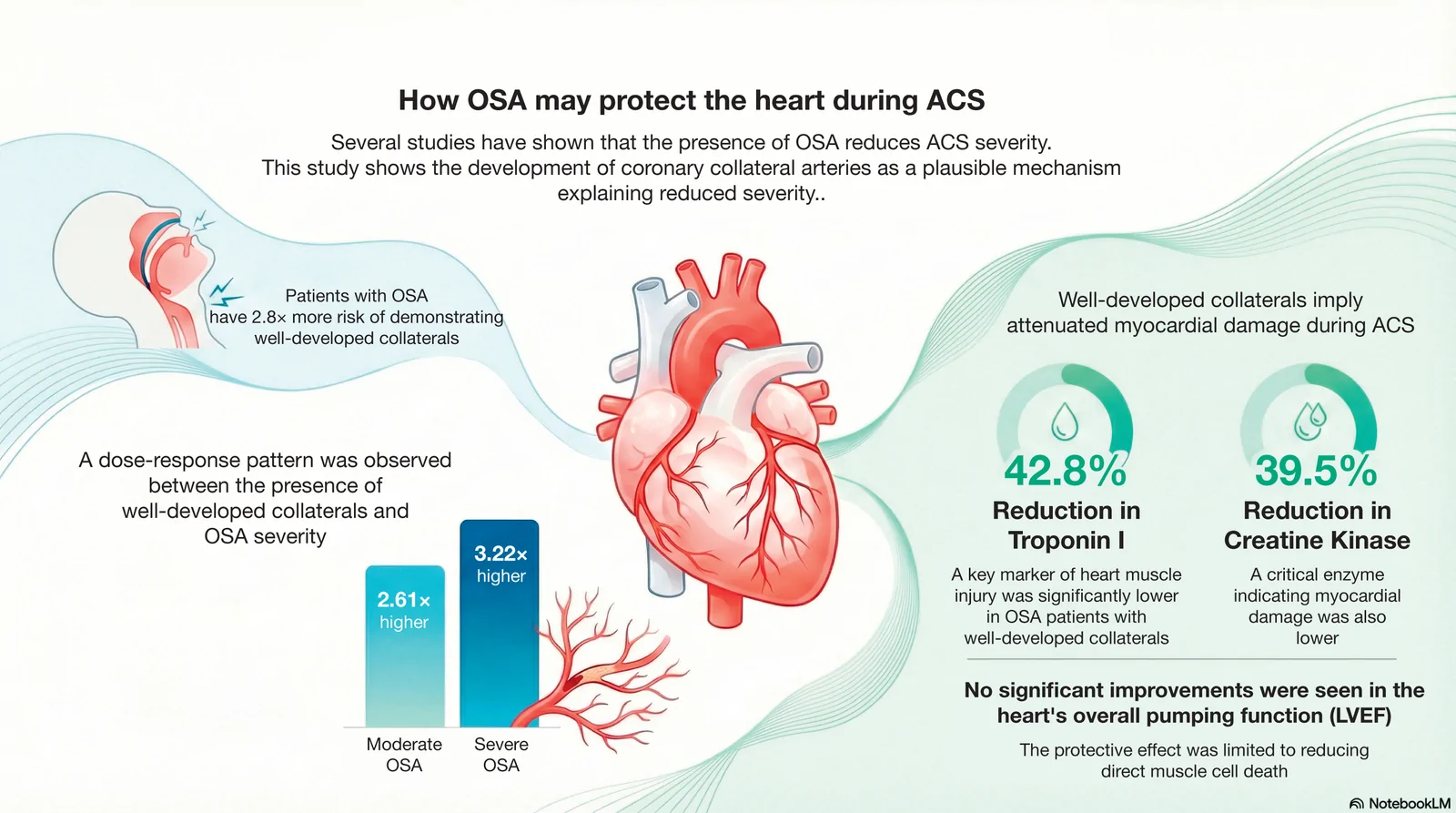
Graphical depiction of the proposed mechanism through which OSA influences ACS event severity. Graphic was generated using NotebookLM. ACS = acute coronary syndrome; LVEF = left ventricular ejection fraction.

A previous study, based on the ISAACC population, showed that patients with ACS and OSA displayed lower peak troponin I levels compared with those without OSA.^5^ Similarly, Allahwala et al^24^ reported lower levels of peak troponin I among patients with STEMI with a history of OSA. Both findings suggest that OSA may have a cardioprotective role in ACS events. Our current study proposes that OSA-driven collateral development could be the main mechanism underlying the reduction in ACS severity (Fig 2). The greater presence of robustly developed collaterals, which has been shown to reduce infarct size,^19^^,^^25^ could explain the lesser extent of myocardial damage observed in the OSA group. The findings of the present study, showing lower cTnI and CK levels among patients with OSA with robustly developed collaterals, are consistent with this evidence.

Although further mechanistic studies are needed to explain the biological pathways that may drive improved collateralization in OSA, our findings add to the growing evidence supporting a potential adaptive vascular response to intermittent hypoxia. This paradoxical cardioprotective adaptation may help to explain the reduced myocardial damage and infarct severity reported in some clinical OSA cohorts.^5^^,^^6^^,^^26^^,^^27^ These results underscore the complex and heterogeneous cardiovascular impact of OSA and highlight the need for future research to determine the clinical relevance of these novel findings for risk stratification and management in ACS populations.

This study includes a larger sample than previous investigations with similar objectives and incorporates measurements of cardiac biomarkers, sleep studies, and coronary angiography performed during the acute phase of ACS. Moreover, it is important to emphasize the value of having a sleep study for the entire cohort, rather than relying solely on clinical history for the diagnosis of OSA, as has been done in previous studies. However, some limitations exist in the present study. First, this study includes primarily male patients, requiring future, larger studies to assess its generalizability to female patients. Evidence indicating sex-specific effects induced by OSA on cardiovascular health exist.^28^^,^^29^ Bouloukaki et al^28^ found that female sex was an independent predictor for the prevalence of cardiovascular disease in a population experiencing OSA-like symptoms. Similarly, Roca et al^29^ showed that levels of some subclinical myocardial injury markers significantly scaled with OSA severity only among female patients. Our predominantly male patient sample could not be used to gauge whether sex-specific OSA effects exist in the prevalence of collaterals or ACS severity. Based on current evidence, future studies explicitly should address a possible differential effect of OSA on ACS severity and collateral prevalence in the female population. Second, the observational nature of the study does not allow inference of causal relationships between OSA and the development of coronary collaterals. Third, ISAACC included only nonsleepy patients with ACS; therefore, more studies are required to extrapolate to a broader population. Finally, sleep studies were performed during or at maximum of 72 hours within the acute phase of the ACS, which may have caused an increase in the amount of central events. However, patients with > 50% central events were excluded from this study.

## Interpretation

In the current study, OSA was associated with the presence of robust coronary collateral circulation among patients with first-time ACS. Moreover, the presence of robust collaterals was associated with a reduction of myocardial damage in patients with OSA, potentially implying improved short-term ACS prognosis in this population. Future mechanistic studies are warranted to corroborate this hypothesis.

## Funding/Support

The ISAACC trial was funded by 10.13039/100017647ResMed (Australia), Fondo de Investigación Sanitaria (Fondo Europeo de Desarrollo Regional), the Spanish Respiratory Society, the Catalonian Cardiology Society, Esteve-Teijin, Oxigen Salud, and Associació Lleidatana de Respiratori (ALLER). The Instituto de Salud Carlos III (ISCIII) is cofunded by the 10.13039/501100000780European Union [Grant PI21/00337]. I. J.-G. was suppported by the ISCIII through a predoctoral fellowship (PFIS: FI24/00084).

## Financial/Nonfinancial Disclosures

The authors have reported to *CHEST* the following: F. B. received a research grant from ResMed (an Australian company that develops products related to sleep apnea), the Health Research Fund, Spanish Ministry of Health, the Spanish Respiratory Society, the Catalonian Cardiology Society, Esteve-Teijin (Spain), Oxigen Salud (Spain), and ALLER to develop the ISAACC trial. ResMed partly funded the study. None declared (K. D., A. A., A. T., M. S.-d.-l.-T., A. S.-d.-l.-T., L. Pinilla, J. A., O. M., L. Pascual, D. M., R. V., I. J.-G., I. D. B.).

## Supplementary Data

e-Online Data
