# Supplementary material for: OSA and Acute Coronary Syndrome Severity: The Potential Role of Coronary Collateral Circulation
Source: Chest. 2026 Mar 7;170(2):543–51. doi: 10.1016/j.chest.2026.02.015 (PMC13494460; doi:10.1016/j.chest.2026.02.015)
Supplement: Supplementary file 1 [file mmc1.docx]

SUPPLEMENTARY APENDIX

## Supplementary methods

## Clinical data collection

Medical history, medication uptake, smoking history and anthropometric variables were recorded during hospital admission. For anthropometric data, patients had their height measured without shoes using a stadiometer attached to a wall, with a precision of 0.5 cm; weight was measured in kg using scales calibrated to manufacturers’ recommendations; waist, neck and hip circumferences were measured whilst unclothed using flexible non-extensible tape calibrated in cm. Waist circumference was taken at the level of the narrowest point between the ribs and the iliac crest. Hip circumference was taken at the level of the greatest posterior protuberance of the buttocks. Neck circumference was taken of the girth around the neck immediately superior to the thyroid cartilage within a subject who was either seated or standing. For biochemical profiling, fasting plasma from all participants was collected in EDTA tubes the day after the sleep study.

**e-Table 1. Comparison of patients included in the analysis vs unincluded ones.**

|  | *All*  *N=478* | *Unincluded*  *n=293* | *Included*  *n=185* | *P* value |
| --- | --- | --- | --- | --- |
| **Sociodemographic** |  |  |  |  |
| Gender |  |  |  | 0.335 |
| Female | 81 (16.9%) | 54 (18.4%) | 27 (14.6%) |  |
| Male | 397 (83.1%) | 239 (81.6%) | 158 (85.4%) |  |
| Age | 58.0 [51.0;66.0] | 59.0 [51.0;67.0] | 57.0 [50.0;65.0] | 0.159 |
| Smoking habits |  |  |  | 0.131 |
| Non-smoker | 98 (20.5%) | 65 (22.2%) | 33 (17.8%) |  |
| Former smoker | 113 (23.6%) | 75 (25.6%) | 38 (20.5%) |  |
| Smoker | 267 (55.9%) | 153 (52.2%) | 114 (61.6%) |  |
| **Anthropometric characteristics** |  |  |  |  |
| BMI (kg/m2) | 28.4 [26.0;31.2] | 28.4 [25.9;30.8] | 28.8 [26.5;31.9] | 0.107 |
| Neck circumference (cm) | 40.0 [38.0;43.0] | 40.0 [38.0;42.0] | 40.0 [38.0;43.0] | 0.589 |
| Waist circumference (cm) | 102 [95.0;110] | 102 [94.0;109] | 103 [95.0;111] | 0.181 |
| Hip circumference (cm) | 102 [97.0;109] | 101 [97.0;108] | 103 [97.0;109] | 0.538 |
| **Comorbidities** |  |  |  |  |
| Hypertension (pre-event): | 261 (54.6%) | 163 (55.6%) | 98 (53.0%) | 0.635 |
| Diabetes mellitus (pre-event): | 112 (23.4%) | 62 (21.2%) | 50 (27.0%) | 0.173 |
| Dyslipidemia (pre-event): | 283 (59.2%) | 176 (60.1%) | 107 (57.8%) | 0.698 |
| **Treatment** |  |  |  |  |
| ACE inhibitors | 119 (24.9%) | 69 (23.6%) | 50 (27.0%) | 0.467 |
| Betablockers | 69 (14.4%) | 49 (16.7%) | 20 (10.8%) | 0.097 |
| ARBs | 64 (13.4%) | 47 (16.1%) | 17 (9.19%) | **0.044** |
| Calcium antagonist | 44 (9.21%) | 34 (11.6%) | 10 (5.41%) | **0.034** |
| Diuretics | 78 (16.3%) | 52 (17.7%) | 26 (14.1%) | 0.349 |
| Hypolipemics | 139 (29.1%) | 87 (29.7%) | 52 (28.1%) | 0.789 |
| **Biochemistry** |  |  |  |  |
| Glucose (mg/dL) | 112 [96.0;150] | 112 [96.0;139] | 115 [96.0;164] | 0.287 |
| Creatinine (mg/dL) | 0.85 [0.73;1.02] | 0.85 [0.72;1.01] | 0.88 [0.75;1.05] | 0.094 |
| Hemoglobin (gr/dL) | 141 [131;151] | 142 [132;152] | 138 [129;149] | 0.059 |
| Total cholesterol (mg/dL) | 181 [155;212] | 182 [158;213] | 176 [155;205] | 0.097 |
| Low-density lipoprotein (mg/dL) | 110 [87.4;136] | 112 [87.6;138] | 107 [88.1;134] | 0.524 |
| High-density lipoprotein (mg/dL) | 41.0 [34.4;47.2] | 41.0 [35.0;48.4] | 41.0 [34.0;46.0] | 0.171 |
| Triglycerides (mg/dL) | 134 [97.0;184] | 134 [95.0;187] | 136 [98.0;181] | 0.906 |
| Platelet (cells/µL) | 204 [172;244] | 202 [170;242] | 208 [178;245] | 0.295 |
| Leukocytes (cells/mm3) | 9.80 [8.10;11.8] | 9.50 [8.00;11.3] | 10.5 [8.47;12.7] | **0.001** |
| **Sleep parameters** |  |  |  |  |
| Apnea-Hypopnea Index (events per hour) | 20.8 [12.0;36.6] | 20.3 [10.0;36.0] | 22.0 [16.0;37.0] | **0.050** |
| Mean SpO2 (%) | 16.7 [7.95;32.5] | 15.3 [6.53;30.8] | 19.6 [11.2;35.9] | 0.309 |
| Minimum SpO2 (%) | 2.00 [0.20;9.38] | 1.50 [0.10;8.97] | 2.90 [0.30;11.3] | 0.552 |
| Time under 90% SpO2 (%) | 93.2 [92.0;94.4] | 93.3 [92.0;94.4] | 93.0 [92.0;94.3] | 0.174 |
| Oxygen Desaturation Index (events per hour) | 85.0 [81.0;88.0] | 85.0 [81.0;88.0] | 85.0 [80.0;88.0] | **0.011** |

Variables are reported as median [Q1; Q3] if continuous and as frequencies (percentage) if categorical. Abbreviations: BMI = Body mass index; ACE = Angiotensin converting enzyme; ARBs = Angiotensin II receptor blockers; SpO2 = peripheral capillary oxygen saturation.

| **e-Table 2.**  **Descriptive of patients with OSA comparing CRS 0/1 and CRS 2/3 groups.** | | | |  |  |
| --- | --- | --- | --- | --- | --- |
|  | *Patients with OSA N=147* | *CRS 0/1*  *N=86* | *CRS 2/3*  *N=61* | *P* value |  |
| **Sociodemographic characteristics** |  |  |  |  |  |
| Gender: |  |  |  | 0.561 |  |
| Men | 126 (85.7%) | 72 (83.7%) | 54 (88.5%) |  |  |
| Women | 21 (14.3%) | 14 (16.3%) | 7 (11.5%) |  |  |
| Age (years) | 57.0 [50.0;64.0] | 57.0 [49.2;63.8] | 58.0 [51.0;64.0] | 0.780 |  |
| Smoking habits: |  |  |  | 0.931 |  |
| Non-smoker | 26 (17.7%) | 16 (18.6%) | 10 (16.4%) |  |  |
| Current smoker | 91 (61.9%) | 53 (61.6%) | 38 (62.3%) |  |  |
| Ex smoker | 30 (20.4%) | 17 (19.8%) | 13 (21.3%) |  |  |
| **Anthropometric characteristics** |  |  |  |  |  |
| BMI (kg/m2) | 29.1 [26.7;32.1] | 29.6 [27.2;32.1] | 28.0 [26.5;31.9] | 0.100 |  |
| Neck circumference (cm) | 40.0 [38.0;43.0] | 40.0 [38.0;43.0] | 40.0 [39.0;42.0] | 0.936 |  |
| Waist circumference (cm) | 103 [96.0;112] | 104 [97.5;112] | 103 [94.0;112] | 0.265 |  |
| Hip circumference (cm) | 103 [97.0;110] | 104 [99.0;112] | 100 [95.0;109] | 0.086 |  |
| **Comorbidities** |  |  |  |  |  |
| Hypertension: | 78 (53.1%) | 45 (52.3%) | 33 (54.1%) | 0.965 |  |
| Diabetes mellitus: | 42 (28.6%) | 23 (26.7%) | 19 (31.1%) | 0.691 |  |
| Dyslipidemia: | 85 (57.8%) | 52 (60.5%) | 33 (54.1%) | 0.548 |  |
| **Treatment** |  |  |  |  |  |
| ACE inhibitor treatment: | 39 (26.5%) | 21 (24.4%) | 18 (29.5%) | 0.618 |  |
| ARB treatment: | 14 (9.52%) | 11 (12.8%) | 3 (4.92%) | 0.188 |  |
| Statin treatment: | 35 (23.8%) | 20 (23.3%) | 15 (24.6%) | 1.000 |  |
| **Admission parameters** |  |  |  |  |  |
| Admitting diagnosis: |  |  |  | 0.061 |  |
| STEMI | 120 (81.6%) | 75 (87.2%) | 45 (73.8%) |  |  |
| NSTEMI | 24 (16.3%) | 9 (10.5%) | 15 (24.6%) |  |  |
| UA | 3 (2.04%) | 2 (2.33%) | 1 (1.64%) |  |  |
| length of hospital stay (days) | 6.00 [5.00;8.00] | 6.00 [4.25;8.00] | 7.00 [5.00;9.00] | 0.063 |  |
| Length of stay in the Cardiology Unit (days) | 2.00 [2.00;3.00] | 2.00 [2.00;2.00] | 2.00 [2.00;3.00] | **0.047** |  |
| **Biochemistry** |  |  |  |  |  |
| Glucose (mg/dL) | 114 [95.5;171] | 115 [98.0;156] | 109 [94.0;182] | 0.709 |  |
| Creatinine (mg/dL) | 0.88 [0.74;1.04] | 0.86 [0.74;1.10] | 0.88 [0.75;0.99] | 0.633 |  |
| Hemoglobin (gr/dL) | 13.8 [12.8;14.9] | 13.6 [12.8;14.6] | 14.0 [13.2;15.1] | 0.130 |  |
| HbA1c (%) | 5.80 [5.50;6.60] | 5.80 [5.50;6.40] | 5.70 [5.50;6.73] | 0.596 |  |
| Total cholesterol (mg/dL) | 179 [157;206] | 179 [156;204] | 178 [159;209] | 0.530 |  |
| Low-density lipoprotein (mg/dL) | 107 [90.0;136] | 104 [86.1;130] | 111 [97.0;138] | 0.073 |  |
| High-density lipoprotein (mg/dL) | 41.0 [34.0;45.8] | 41.0 [34.0;46.6] | 37.0 [33.0;43.0] | 0.203 |  |
| Triglycerides (mg/dL) | 141 [102;184] | 138 [99.5;189] | 146 [110;178] | 0.849 |  |
| Platelet (cells/µL) x 10³ | 209 [180;246] | 208 [176;234] | 215 [190;254] | 0.179 |  |
| Leukocytes (cells/mm3) x 10³ | 10.3 [8.40;12.6] | 10.4 [8.93;12.4] | 10.0 [7.90;12.8] | 0.377 |  |
| Neutrophils (%) | 77.0 [68.2;83.0] | 77.5 [70.0;84.0] | 76.0 [67.5;82.0] | 0.256 |  |
| **Sleep parameters** |  |  |  |  |  |
| Apnea-Hypopnea Index (events per hour) | 26.8 [19.4;41.7] | 26.8 [19.7;44.3] | 26.9 [19.3;39.5] | 0.861 |  |
| Oxygen Desaturation Index (events per hour) | 24.4 [15.0;39.9] | 23.6 [15.3;41.2] | 26.4 [13.0;38.7] | 0.766 |  |
| Time under 90% SpO2 (%) | 3.90 [0.60;16.1] | 4.20 [0.60;17.2] | 3.10 [0.45;9.25] | 0.336 |  |
| Mean SpO2 (%) | 93.0 [91.9;94.2] | 92.9 [91.6;94.0] | 93.4 [92.0;94.3] | 0.142 |  |
| Minimum SpO2 (%) | 84.0 [79.0;87.0] | 84.0 [79.0;87.0] | 84.5 [79.8;87.2] | 0.756 |  |
| OSA categories |  |  |  | 0.735 |  |
| Moderate OSA | 85 (57.8%) | 51 (59.3%) | 34 (55.7%) |  |  |
| Severe OSA | 62 (42.2%) | 35 (40.7%) | 27 (44.3%) |  |  |
| **Cohen Rentrop Scale distribution** |  |  |  |  |  |
| Degree of coronary collateral circulation (Rentrop): |  |  |  | **<0.001** |  |
| Rentrop 0 | 58 (39.5%) | 58 (67.4%) | 0 (0.00%) |  |  |
| Rentrop 1 | 28 (19.0%) | 28 (32.6%) | 0 (0.00%) |  |  |
| Rentrop 2 | 33 (22.4%) | 0 (0.00%) | 33 (54.1%) |  |  |
| Rentrop 3 | 28 (19.0%) | 0 (0.00%) | 28 (45.9%) |  |  |
| Variables are reported as median [Q1; Q3] if continuous and as frequencies (percentage) if categorical. Differences between groups were tested with Wilcoxon-ranked sum test if variables were continuous and Fisher’s exact test if they were categorical. Abbreviations: BMI = Body mass index; ACE = angiotensin II converting enzyme; ARB =Angiotensin II receptor blockers; STEMI = Segment T elevation myocardial infarction; NSTEMI = Non-segment T elevation myocardial infarction; UA = Unstable angina; HbAc1 = glycated hemoglobin; SpO2 = peripheral capillary oxygen saturation. | | | | |  |
|  |  |  |  |  |  |

**e-Figure 1. Flowchart of sample selection for the study.**

ACS = Acute coronary syndrome; CCC = Coronary collateral circulation; OSA = Obstructive sleep apnea. OSA was defined based on respiratory polygraphy determined apnea-hypopnea index (AHI). An AHI ≥ 15 events/hour was considered OSA.

**e-Figure 2. Association between apnea-hypopnea index and collaterality.**

Plot presents the probability for Cohen Rentrop Scales of 2 or 3 (CRS 2/3) using apnea-hypopnea index (AHI) as the predictor variable. Predictions were computed using a generative additive model with AHI data from the 185 patients in the study population. The X axis represents the theoretical AHI value and the Y axis the predicted probability of CRS 2/3. The red line represents the predicted probability of risk CRS 2/3 and the gray area represents the confidence intervals.

**e-Figure 3. Association between sleep-apnea specific hypoxic burden and collaterality.**

Plot presents the probability for Cohen Rentrop Scales of 2 or 3 (CRS 2/3) using sleep apnea-hypopnea specific hypoxic burden (HB) as the predictor variable. Predictions were computed using a generative additive model with HB data from the 129 patients with available HB records (24 non-OSA and 105 OSA patients). The X axis represents the theoretical HB value and the Y axis the predicted probability of CRS 2/3. The red line represents the predicted probability of risk CRS 2/3 and the gray area represents the confidence intervals.
